# Building for the future

**DOI:** 10.7554/eLife.00856

**Published:** 2013-05-28

**Authors:** Hugh Pelham

**Affiliations:** MRC Laboratory of Molecular Biology, Cambridge, United Kingdomhp@mrc-lmb.cam.ac.uk

**Keywords:** point of view, science policy, history of science, MRC, LMB, careers in science, funding

## Abstract

As the staff of the MRC Laboratory of Molecular Biology settle into their new building in Cambridge, its director **Hugh Pelham** explains the challenges of living up to its prestigious past.

The MRC Laboratory of Molecular Biology (LMB) got off to a flying start. It opened in early 1962, bringing together groups headed by Max Perutz, Aaron Klug and Fred Sanger, who was already a Nobel laureate for his work on the structure of proteins, notably insulin. By the end of the year it was home to another three laureates: Perutz and his colleague John Kendrew received the chemistry prize for their work on the structure of globular proteins, while Francis Crick shared the physiology or medicine prize with James Watson (who had been in the old MRC Unit for Molecular Biology with Crick, Kendrew and Perutz) and Maurice Wilkins for their work on the structure of DNA.

Half a century and another nine laureates later, as the Medical Research Council (MRC) celebrates its centenary and the LMB moves into a new purpose-built building (Figure 1), it is timely to reflect on how the laboratory works and what key features are worth preserving.

**Figure 1.**
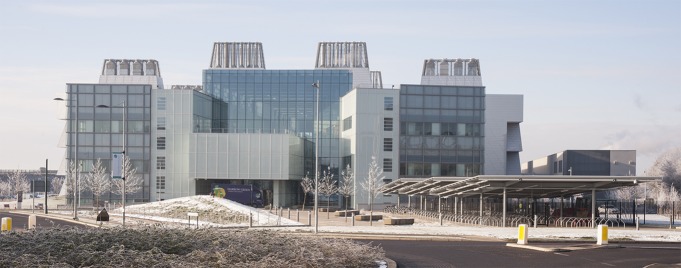
The MRC Laboratory of Molecular Biology (LMB) has moved into a new building, close to the original LMB building (not shown), on the site of Addenbrookes Hospital on the outskirts of Cambridge. The new building, constructed at a cost of £212m, is twice as large as the original building, with greatly improved facilities. Nine Nobel prizes have been shared among 13 LMB scientists.

PHOTOGRAPH: MRC LABORATORY OF MOLECULAR BIOLOGY

It is important that the LMB has a history. This is not because we pay constant homage to the Nobel laureates of the past, although they are hard to forget, but because the founders of the LMB, with the encouragement of the MRC, created a remarkably successful style and culture that is much easier to maintain than to create from scratch. This culture unequivocally values science over all else, and everyone at the LMB, whatever their role, accepts that their goal is to make it as easy as possible for great science to get done. In turn, there is a strong expectation that ambitious, important and long-term problems are tackled.

Perhaps the single most important factor that distinguishes the LMB from a university department, and from some research institutes, is that over 80% of the funding comes from a single source (the MRC). Moreover, this core funding is for a period of five years (and amounts to almost £170m for the period 2012-2017).

Although this core funding is for the purpose of pursuing an agreed strategy, we have considerable flexibility in how it is used: in particular, we do not give rigid budgets to individual groups, which means that we can allocate resources as and when the science requires. This also allows us to exploit unexpected opportunities. And because the budget depends on the overall performance of the LMB, which is reviewed every five years by the MRC, it is in everyone's interest that their colleagues do well. Coupled with the fact that the majority of equipment is communal, this model has created a tradition of freely offered help, advice, reagents, ideas and facilities, which most LMB scientists simply take for granted.

## Flexible working

The LMB is divided into four divisions—cell biology, neurobiology, protein and nucleic acid chemistry, and structural studies—and the heads of those divisions, together with the director, have the responsibility and power to make strategic choices. The passage of time has shown the wisdom of some of the choices made in the past. For example, when Greg Winter was being considered for tenure at the LMB in the mid-1980s, Fred Sanger, who was head of the protein and nucleic acid chemistry division at the time, suggested that he would fit into the division better if he applied his newly-developed protein engineering methods to antibodies rather than enzymes. Winter went on to humanize monoclonal antibodies, a breakthrough that has revolutionized medicine (and has also led to three highly successful biotech companies).

Sometimes, the strategy to follow is obvious because ambitious goals can take over twenty years, a great deal of persistence, to achieve. In addition to Winter's work on antibodies, other examples include the ongoing analysis of the nematode nervous system, and work on the structures of G-protein coupled receptors, which had its origins in early studies of bacteriorhodopsin. Again, history can be important: the LMB pioneered the use of both X-ray crystallography and electron microscopy to determine the structure of proteins, and we remain committed to being world-leading in the determination of large structures.

But the principle established early on was that the best way to make breakthroughs is to hire the brightest scientists possible and let them determine what to do. They are better placed than anyone to work out where the opportunities lie, and some of the greatest discoveries come from unexpected angles. Central funding and flexibility also mean that risk taking can be encouraged, and serendipity exploited.

In general, guided by the broader strategic goals of the MRC, we try to build on our strengths and to have groups with complementary but related interests. Nevertheless, when selecting new group leaders, we look for bright, flexible and imaginative scientists; these qualities are more important to us than the precise area in which they have most recently worked. Most of our recruitment is at the level of young scientists just starting a group, but more senior scientists are also attracted by the LMB ethos: for example, Venki Ramakrishnan, who shared the Nobel Prize in Chemistry in 2009 for his work on the ribosome, moved from the US to the LMB because it offered the long-term support and relative security that he felt was needed to tackle a problem as ambitious as determining the structure of the ribosome.

In such an environment, the greatest pressure to do excellent, relevant and important work comes from one's peers. We encourage as much interaction as possible, and a conversation over coffee or feedback from an internal seminar can be as powerful an influence as any instruction from on high. Certainly as a young group leader myself, my greatest ambition was to be taken seriously by the intimidating intellectuals around the lab, who were very accessible and took an alarmingly close interest in what I was doing.

Though self-motivation is the rule, it can of course be steered both by explicit encouragement and by more subtle influences. It is no coincidence that the LMB is located next to Addenbrookes Hospital, or that it has contacts with industry. Indeed, we are building a closer relationship with Cambridge University School of Clinical Medicine by housing part of the Department of Medicine within our new building, and we have also space set aside for temporary translational projects and collaborations. Our experience is that the most successful collaborations arise though the active encouragement of willing partners, rather than any compulsion.

## Inside the new LMB

In designing the new building, we sought to retain and enhance the most important features of the old LMB, including workshop facilities and a rooftop restaurant, while increasing the space available for specialist equipment and making it easier to maintain and remodel the building without disrupting the researchers working in it.

**Figure 2.**
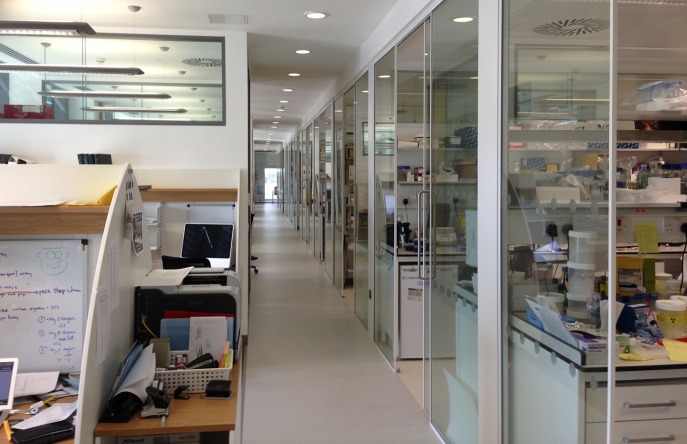
A corridor in the new LMB building, with labs on the right and write-up spaces and offices on the left. The building is designed to encourage interactions between researchers in the corridors and elsewhere; such interactions were a defining characteristic of the original LMB building.

We started with the benches and worked outwards—which was undoubtedly the best way to ensure that the building serves the needs of the scientists. To fit the LMB's hands-on style, there are write-up spaces close to the benches, and offices of group leaders are intermingled with the write-up spaces (Figure 2). The labs are partially divided, making it easy to move between them, to share equipment and to have groups that fluctuate in size. Equipment rooms are across a corridor from the labs and benches, making them accessible to all and creating a space for people to bump into each other: indeed, we took a conscious decision to make the density of scientists on each corridor the same as that in the first LMB building.

PHOTOGRAPH: MRC LABORATORY OF MOLECULAR BIOLOGY

**Figure 3.**
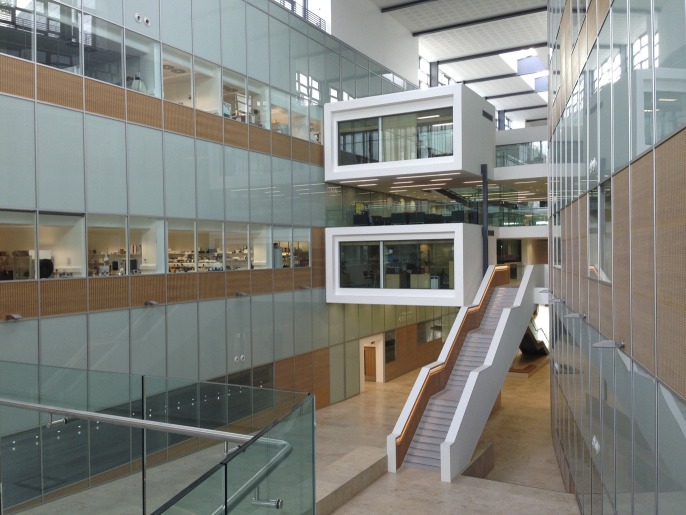
The central atrium of the new LMB building. There are three floors of labs and offices, with services such as air ducts being located between the floors. The two white structures to the left of the staircase each house a meeting room and an office.

We also wanted a building that was easy to navigate, and in which it was easy to find people. Windows into labs and equipment rooms, a central atrium that acts as a ‘street’ (Figure 3), and breakout areas that are quiet (but visible) have enabled us to achieve this. The end result is a building carefully adapted to the culture of the LMB, with a particular emphasis on interaction and communal sharing. And although the new building is twice as large as the previous one, it feels more coherent.

PHOTOGRAPH: MRC LABORATORY OF MOLECULAR BIOLOGY

Behind the scenes, we paid particular attention to the services in the building and their future flexibility. Unusually for a European building, there are full-height interstitial zones between the floors, which mean that the air ducts, pipework and wiring can all be accessed for maintenance and modification. Elsewhere, the main air handling units and other plant are not on the roof, as they usually are, but in towers adjacent to the building. This takes weight and vibration away from the lab structure and also creates iconic stainless steel-clad features to bemuse the onlooker.

## Challenges for the future

A pressing question in these hard economic times is whether the LMB style of science, with its need for relatively secure, long-term funding, will continue to be supported as governments seek quantifiable and preferably rapid returns on their investments in research. The latter approach tends to result in the avoidance of risk and the setting of defined targets, making it largely incompatible with difficult, long-term projects. Although grants can focus funding on particular projects, institutes that rely on grants for overheads cannot have the same level of flexibility and freedom, and may struggle when grants dry up.

At the LMB we would argue, as the evidence of the past supports, that core funding of long-term ambitious projects decided upon by scientists is still worthwhile. We also firmly believe that such funding will create an environment that is attractive to industry, will train the world's best scientists for the future, and will directly or indirectly generate discoveries, applications, medical advances and wealth. Some of the major research charities, notably the Howard Hughes Medical Institute and the Wellcome Trust, recognize the need for longer term, more flexible funding and they have tailored their support accordingly, empowering the best scientists to be bold. The LMB has always done this.

The challenge for the LMB is to maintain scientific quality in the face of ever-increasing competition and, at the same time, to preserve a distinctive way of doing science. We now have an excellent building in which to operate, and every reason to work hard.

